# Maternal Eating Disorders, Body Mass Index, and Offspring Psychiatric Diagnoses

**DOI:** 10.1001/jamanetworkopen.2024.40517

**Published:** 2024-10-22

**Authors:** Ida A. K. Nilsson, Judit Ozsvar, Mika Gissler, Catharina Lavebratt

**Affiliations:** 1 Department of Molecular Medicine and Surgery, Karolinska Institutet, Stockholm, Sweden; 2 Center for Molecular Medicine, Karolinska University Hospital, Stockholm, Sweden; 3 Centre for Eating Disorders Innovation, Karolinska Institutet, Stockholm, Sweden; 4 Department of Knowledge Brokers, Finnish Institute for Health and Welfare, Helsinki, Finland; 5 Research Centre for Child Psychiatry, University of Turku, Turku, Finland

## Abstract

**Importance:**

Maternal nutrition is essential in fetal development; thus, disordered eating may influence this process and contribute to the development of offspring psychiatric disorders.

**Objective:**

To investigate the association of maternal eating disorders and prepregnancy body mass index (BMI) with offspring psychiatric diagnoses.

**Design, Setting, and Participants:**

This population-based cohort study used Finnish national registers to assess all live births from January 1, 2004, through December 31, 2014, with follow-up until December 31, 2021. The data analyses were conducted from September 1, 2023, to September 30, 2024.

**Exposures:**

Maternal eating disorder and prepregnancy BMI.

**Main Outcomes and Measures:**

Primary outcomes were 9 neurodevelopmental and psychiatric offspring diagnoses. Cox proportional hazards modeling adjusted for potential risk factors in the development of the outcome disorders was applied in 2 models. Secondary analyses were stratified for adverse birth outcomes (prematurity, small size for gestational age, and low Apgar score) or comorbid offspring eating disorders. Categories of BMI (calculated as weight in kilograms divided by height in meters squared) included underweight (BMI <18.5), normal weight (18.5-24.9), overweight (25.0-29.9), obesity (30.0-34.9), and severe obesity (≥35.0).

**Results:**

The mean (SD) age of 392 098 included mothers was 30.15 (5.38) years, 42 590 mothers (10.86%) were born outside of Finland, 6273 mothers (1.60%) had a history of an eating disorder, 23 114 mothers (5.89%) had prepregnancy underweight, and 208 335 (53.13%) mothers had overweight or obesity. Among 649 956 included offspring, 332 359 (51.14%) were male, and 106 777 (16.43%) had received a neurodevelopmental or psychiatric diagnosis. Maternal eating disorders, prepregnancy underweight, and overweight or obesity were associated with most of the studied mental diagnoses in offspring, even after adjusting for potential covariates. The largest effect sizes were observed for maternal eating disorders not otherwise specified in association with offspring sleep disorders (hazard ratio [HR], 3.34 [95% CI, 2.39-4.67]) and social functioning and tic disorders (HR, 2.79 [95% CI, 2.21-3.52]), while for maternal severe prepregnancy obesity, offspring intellectual disabilities (HR, 2.04 [95% CI, 1.83-2.28]) had the largest effect size. Adverse birth outcomes further increased the risk of offspring having other feeding disturbances of childhood and infancy (eg, HR, 4.53 [95% CI, 2.97-6.89] for maternal eating disorders) and attention-deficit/hyperactivity disorder and conduct disorder (eg, HR, 2.27 [95% CI, 1.74-2.96] for maternal anorexia nervosa).

**Conclusions and Relevance:**

In this population-based cohort study including 392 098 mothers and 649 956 offspring, offspring from mothers with an eating disorder history or prepregnancy BMI outside normal weight were at higher risk of psychiatric disorders. The results differed somewhat between the 2 exposures with regard to which offspring diagnoses had associations, and effect sizes were typically larger for maternal eating disorders vs BMI. These findings suggest a need to consider these 2 exposures clinically to help prevent offspring mental illness.

## Key Points

**Question:**

Are maternal eating disorders or body mass index (BMI) associated with offspring psychiatric diagnoses?

**Findings:**

In this cohort study assessing data from 392 098 mothers and 649 956 offspring, there were associations between maternal history of eating disorders and prepregnancy BMI outside normal weight with most of the 9 studied psychiatric diagnoses in offspring. Effect sizes were typically larger for maternal eating disorders vs BMI.

**Meaning:**

The findings underline the risk of offspring mental illness associated with maternal eating disorders and prepregnancy BMI and suggest the need to consider these exposures clinically to help prevent offspring mental illness.

## Teaser

This cohort study examines whether there is an association between maternal eating disorders or prepregnancy body mass index and psychiatric diagnoses in offspring.

## Introduction

Fetal neurodevelopment is a highly regulated and sensitive process influenced by internal and external factors. The nutritional status prior to and during pregnancy is critical for the developing brain^1,2^ and macronutrient and micronutrient deficiencies during pregnancy may disturb these processes. Studies show associations between particular deficiencies and offspring psychiatric disorders, including maternal vitamin D deficiency and autism spectrum disorder (ASD), attention-deficit/hyperactivity disorder (ADHD), and schizophrenia,^3,4^ low or high maternal serum vitamin B_12_ levels and ASD,^5^ and maternal iron deficiency and schizophrenia.^6^ Eating disorders commonly result in nutritional deficiencies.^7^ Similar to malnourishment in pregnancy,^1,8,9^ maternal eating disorders have a detrimental effect on pregnancy, delivery, and neonatal outcomes.^10,11,12^ Different maternal eating disorders may influence different offspring psychopathologies. Ongoing and previous maternal anorexia nervosa (AN) has been associated with offspring ADHD and ASD, with higher risk observed for active AN.^13^ Maternal history of AN has been associated with offspring emotional disorders, in particular anxiety and depression.^14,15^ Exposure to lifetime maternal bulimia nervosa (BN) increases the incidence of offspring obsessive-compulsive disorder symptoms, ADHD, ASD, and emotional disorders,^13,14,15,16^ whereas lifetime maternal binge eating disorder is associated with emotional, affective, and anxiety disorders in offspring.^17,18^ In addition, maternal obesity has repeatedly been associated with the risk of psychiatric disorders, such as ASD and ADHD in offspring.^19,20,21,22,23,24,25^ Maternal famine, on the other hand, has been associated with a range of mental disorders, including schizophrenia,^26^ affective disorders,^27,28^ antisocial personality disorder,^29^ and depressive symptoms.^30^ Furthermore, increased incidence of nonaffective psychosis in adolescence exposed to maternal underweight or inadequate weight gain in early pregnancy has been reported.^31^ Thus, maternal body mass index (BMI) and eating disorders play a significant role in offspring mental health, but the link with, in particular, eating disorder not otherwise specified (EDNOS), is not well investigated. Moreover, eating disorders include more than deviations in BMI, and pathological mechanisms are believed to differ from those of other disorders with altered BMI or, for example, constitutional leanness. In addition, AN and eating disorders in general are commonly accompanied by, for example, psychiatric comorbidities, dietary restriction, and extreme physical activity. Contrary to individuals with AN, most individuals with other eating disorders are not underweight, but still have significant somatic complications and psychiatric comorbidities.^32^ Here, we sought to expand existing knowledge on associations of different maternal eating disorders and prepregnancy BMI with neurodevelopmental and psychiatric diagnoses in offspring. We also explored differences between exposure to maternal AN vs maternal underweight with offspring mental health.

## Methods

### Study Population

This population-based register cohort study used data from the Finnish Medical Birth Register, the Finnish Care Register for Health Care (HILMO), and the Finnish Register on Reimbursement Drugs (RRD). This study included all live births in Finland between January 1, 2004, and December 31, 2014, followed up until December 31, 2021. The data analyses were conducted from September 1, 2023, to September 30, 2024. Information on maternal and offspring diagnoses were obtained from HILMO using the *International Classification of Diseases, Ninth Revision* (*ICD-9;* 1987-1995), and *Tenth Revision* (*ICD-10*; since 1996). Data on purchases of reimbursed medications were obtained using Anatomical Therapeutic Chemical codes from the RRD. Register data were linked using the encrypted personal identification numbers assigned to all Finnish citizens and permanent residents. The use of register data from the Social Insurance Institution and the Finnish Institute for Health and Welfare and linkages between them was carried out after receiving the necessary permission from the Finnish Social and Health Data Permit Authority (Findata) and the Swedish Ethics Review Authority. All procedures contributing to this work complied with the ethical standards of the relevant national and institutional committees on human experimentation and with the Helsinki Declaration of 1975, as revised in 2008. The data were pseudonymized by Findata and analyzed in the secured environment Kapseli provided by Findata. Findata and the Swedish Ethics Review Authority indicated that informed consent was not required since the study material was based on register data only and no registered persons were contacted. The researchers had no access to personal identifiable information. This study follows the Strengthening the Reporting of Observational Studies in Epidemiology (STROBE) reporting guideline.

### Outcomes

Data on the diagnoses of the offsprings’ neurodevelopmental or psychiatric disorders were collected from HILMO. The diagnosis status was identified and grouped by the *ICD-10* codes of any mental, behavioral, and neurodevelopmental disorder (F00-F99); mood disorders (F30-39, F92); anxiety disorders (F40-43, F93); sleep disorders (F51); intellectual disabilities (F70-79); specific developmental disorders (F80-83); ASD (F84); ADHD and conduct disorders (F90-91); disorders of social functioning with onset specific to childhood and adolescence, including selective mutism and attachment disorders, and tic disorders and Tourette syndrome (hereafter called social functioning and tic disorders) (F94-95); and other feeding disorders of infancy and childhood (F98.2).

### Main Exposures

Maternal prepregnancy BMI was calculated as weight in kilograms divided by height in meters squared using maternal height and prepregnancy weight reported from the first maternal care visit in the Medical Birth Register. It was categorized according to the World Health Organization guidelines as underweight for BMI less than 18.5; normal weight, 18.5 to 24.9; overweight, 25.0 to 29.9; obesity, 30.0 to 34.9; and severe obesity, 35.0 or higher. The data on maternal eating disorder diagnoses were obtained from HILMO, all diagnosed before pregnancy and identified using *ICD* codes as follows: any maternal eating disorder (*ICD-9*, 3071A, 3075B, and 3075E; *ICD-10*, F50), AN (*ICD-9*, 3071A; *ICD-10*, F50.0, F50.1), BN (*ICD-9*, 3071B; *ICD-10*, F50.2, F50.3), and EDNOS (*ICD-9*, 3071E; *ICD-10*, F50.8, F50.9).

### Covariates

The covariates were selected with regard to their possible association with the development of psychiatric disorders.^33,34,35,36,37,38,39,40,41,42^ The following covariates with categories as described in the Table were taken into consideration in adjusted model 1: offspring birth year and sex, number of fetuses, maternal age group at delivery, parity, unmarried mother at birth, mother’s country of birth, maternal smoking during pregnancy, and maternal socioeconomic status. For maternal eating disorders as an exposure, we also included maternal BMI as a covariate in model 1, while for maternal BMI, we included outpatient psychiatric disorder in mothers before or during pregnancy (*ICD-9*, 290-319; *ICD-10*, F00-F99). In adjusted model 2, we added information on maternal systemic or bowel inflammatory disorder (*ICD-10*, M30-M36, K50, K51, and K52.3) and maternal diabetes before or during pregnancy obtained from HILMO to the covariates in model 1. Diabetes diagnoses (*ICD-10*, E11, E14, O24.1, and O24.4) were obtained from HILMO, and maternal insulin-treated pregestational diabetes was obtained from the RRD.

**Table.** Frequencies of Exposures, Covariates, and Adverse Birth Outcomes in the Cohort, by Offspring Psychiatric Diagnosis

Maternal variable	Offspring, No. (%)										
	Total cohort	Any neurodevelopmental or psychiatric disorder	Mood disorders	Anxiety disorders	Other feeding disturbances of infancy and childhood	Sleep disorders	Intellectual disabilities	Specific developmental disorders	ASD	ADHD and conduct disorders	Social functioning and tic disorders
Total, No.^a^	649 956	106 777	19 641	33 687	3700	3753	5684	41 570	9659	32 242	8523
Exposure^b^											
Maternal prepregnancy BMI category^c^											
Underweight	23 114 (3.56)	4221 (3.95)	722 (3.68)	3748 (11.13)	148 (4.00)	129 (3.44)	252 (4.43)	1731 (4.16)	365 (3.78)	1283 (3.98)	385 (4.52)
Normal weight	384 811 (59.21)	56 949 (53.33)	9922 (50.52)	17 804 (52.85)	2155 (58.24)	2143 (57.10)	2795 (49.17)	21 032 (50.59)	5075 (52.54)	16 697 (51.79)	4696 (55.10)
Overweight	134 584 (20.71)	22 675 (21.23)	4025 (20.49)	6874 (20.41)	746 (20.16)	814 (21.69)	1275 (22.43)	9139 (21.98)	2063 (21.36)	6999 (21.71)	1673 (19.63)
Obesity	49 909 (7.68)	9869 (9.24)	1684 (8.57)	2925 (8.68)	316 (8.54)	281 (7.49)	632 (11.12)	4331 (10.42)	992 (10.27)	3286 (10.19)	781 (9.16)
Severe obesity	23 842 (3.67)	5502 (5.15)	1000 (5.09)	1600 (4.75)	162 (4.38)	133 (3.54)	391 (6.88)	2600 (6.25)	549 (5.68)	2026 (6.28)	403 (4.73)
Missing	33 696 (5.18)	7561 (7.08)	2288 (11.65)	3149 (9.35)	173 (4.68)	253 (6.74)	339 (5.96)	2737 (6.58)	615 (6.37)	1951 (6.05)	585 (6.86)
Maternal eating disorders, before pregnancy											
No	643 683 (99.03)	105 391 (98.70)	19 410 (98.82)	33 230 (98.64)	3643 (98.46)	3674 (97.90)	5628 (99.01)	41 099 (98.87)	9519 (98.55)	31 726 (98.40)	8351 (97.98)
Any	6273 (0.97)	1386 (1.30)	231 (1.18)	457 (1.36)	57 (1.54)	79 (2.10)	56 (0.99)	471 (1.13)	140 (1.45)	516 (1.60)	172 (2.02)
AN	3271 (0.50)	673 (0.63)	104 (0.53)	219 (0.65)	23 (0.62)	37 (0.99)	26 (0.46)	252 (0.61)	66 (0.68)	229 (0.71)	88 (1.03)
BN	2574 (0.40)	591 (0.55)	110 (0.56)	198 (0.59)	28 (0.76)	33 (0.88)	18 (0.32)	167 (0.40)	62 (0.64)	241 (0.75)	81 (0.95)
EDNOS	2005 (0.31)	517 (0.48)	88 (0.45)	178 (0.53)	20 (0.54)	35 (0.93)	19 (0.33)	190 (0.46)	53 (0.55)	211 (0.65)	72 (0.84)
Covariates^b^											
Birth year											
2004	57 657 (8.86)	13 646 (12.78)	4752 (24.19)	6457 (19.17)	289 (7.81)	504 (13.43)	655 (11.52)	3982 (9.58)	1108 (11.47)	3425 (10.62)	869 (10.20)
2005	57 634 (8.87)	12 885 (12.07)	3952 (20.12)	5686 (16.88)	288 (7.78)	460 (12.26)	626 (11.01)	4036 (9.71)	1050 (10.87)	3424 (10.62)	915 (10.74)
2006	58 859 (9.06)	12 160 (11.39)	3348 (17.05)	5002 (14.84)	385 (10.41)	393 (10.47)	566 (9.96)	3939 (9.48)	1008 (10.44)	3401 (10.55)	892 (10.47)
2007	58 726 (9.04)	10 768 (10.08)	2410 (12.27)	3920 (11.64)	368 (9.95)	316 (8.42)	582 (10.24)	3821 (9.19)	1031 (10.67)	3272 (10.15)	900 (10.56)
2008	59 605 (9.17)	10 101 (9.46)	1713 (8.72)	3347 (9.94)	329 (8.89)	304 (8.10)	554 (9.75)	4003 (9.63)	1022 (10.58)	3294 (10.22)	901 (10.57)
2009	60 581 (9.32)	9558 (8.95)	1200 (6.11)	2707 (8.04)	324 (8.76)	295 (7.86)	513 (9.03)	3990 (9.60)	968 (10.02)	3279 (10.17)	897 (10.52)
2010	61 191 (9.41)	9027 (8.45)	808 (4.11)	2170 (6.44)	344 (9.30)	305 (8.13)	542 (9.54)	3922 (9.43)	878 (9.09)	3272 (10.15)	891 (10.45)
2011	60 092 (9.25)	8289 (7.76)	621 (3.16)	1680 (4.99)	397 (10.73)	287 (7.65)	473 (8.32)	3732 (8.98)	789 (8.17)	2952 (9.16)	711 (8.34)
2012	59 689 (9.18)	7563 (7.08)	408 (2.08)	1252 (3.72)	311 (8.41)	279 (7.43)	415 (7.30)	3581 (8.61)	710 (7.35)	2526 (7.83)	665 (7.80)
2013	58 373 (8.98)	6907 (6.47)	278 (1.42)	899 (2.67)	355 (9.59)	310 (8.26)	406 (7.14)	3406 (8.19)	580 (6.00)	2032 (6.30)	541 (6.35)
2014	57 639 (8.87)	5873 (5.50)	151 (0.77)	567 (1.68)	310 (8.38)	300 (7.99)	352 (6.19)	3158 (7.60)	515 (5.33)	1365 (4.23)	341 (4.00)
Sex											
Female	317 597 (48.86)	43 299 (40.55)	10 726 (54.61)	18 656 (55.38)	1827 (49.38)	1622 (43.22)	2017 (35.49)	12 833 (30.87)	2457 (25.44)	8133 (25.22)	2942 (34.52)
Male	332 359 (51.14)	63 478 (59.45)	8915 (45.39)	15 031 (44.62)	1873 (50.62)	2131 (56.78)	3667 (64.51)	28 737 (69.13)	7202 (74.56)	24 109 (74.78)	5581 (65.48)
No. of fetuses											
1	631 249 (97.12)	103 262 (96.71)	19 189 (97.70)	32 846 (97.50)	3538 (95.62)	3540 (94.32)	5470 (96.24)	39 874 (95.92)	9410 (97.42)	31 285 (97.03)	8294 (97.31)
2	18 353 (2.82)	3431 (3.21)	446 (2.27)	825 (2.45)	156 (4.22)	200 (5.33)	208 (3.66)	1655 (3.98)	244 (2.53)	940 (2.92)	224 (2.63)
≥3	354 (0.05)	84 (0.08)	6 (0.03)	16 (0.05)	6 (0.16)	11 (0.29)	6 (0.11)	41 (0.10)	5 (0.05)	16 (0.05)	5 (0.06)
Maternal age, y											
<20	15 213 (2.34)	4430 (4.15)	1177 (5.99)	1662 (4.93)	105 (2.84)	109 (2.90)	212 (3.73)	1673 (4.02)	308 (3.19)	1863 (5.78)	460 (5.40)
20-24	100 613 (15.48)	20 777 (19.46)	4464 (22.73)	6804 (20.20)	616 (16.65)	615 (16.39)	1042 (18.33)	8129 (19.55)	1660 (17.19)	7497 (23.25)	1673 (19.63)
25-29	205 481 (31.61)	32 482(30.42)	5868 (29.88)	9984 (29.64)	1151 (31.11)	1115 (29.71)	1651 (29.05)	12 309 (29.61)	3011 (31.17)	9982 (30.96)	2597 (30.47)
30-34	205 412 (31.60)	29 770 (27.88)	4871 (24.80)	9123 (27.08)	1116 (30.16)	1159 (30.88)	1542 (27.13)	11 449 (27.54)	2734 (28.31)	8044 (24.95)	2279 (26.74)
≥35	123 233 (18.96)	19 318 (18.09)	3261 (16.60)	6114 (18.15)	712 (19.24)	755 (20.12)	1237 (21.76)	8010 (19.27)	1946 (20.15)	4856 (15.06)	1514 (17.76)
Missing	4 (0)	0 (0)	0 (0)	0 (0)	0 (0)	0 (0)	0 (0)	0 (0)	0 (0)	0 (0)	0 (0)
Parity											
0	271 739 (41.81)	47 547 (44.53)	8670 (44.14)	15 102 (44.83)	1835 (49.59)	1782 (47.48)	2292 (40.32)	17 106 (41.15)	5214 (53.98)	15 205 (47.16)	4236 (49.70)
1	218 314 (33.59)	33 176 (31.07)	6273 (31.94)	10 408 (30.90)	1151 (31.11)	1272 (33.89)	1734 (30.51)	12 843 (30.89)	2596 (26.88)	9760 (30.27)	2446 (28.70)
2	95 532 (14.70)	15 429 (14.45)	2849 (14.51)	4821 (14.31)	443 (11.97)	423 (11.27)	900 (15.83)	6647 (15.99)	1142 (11.82)	4441 (13.77)	1075 (12.61)
3	33 174 (5.10)	5857 (5.49)	1089 (5.54)	1893 (5.62)	156 (4.22)	155 (4.13)	365 (6.42)	2706 (6.51)	389 (4.03)	1663 (5.16)	456 (5.35)
≥4	30 701 (4.72)	4673 (4.37)	736 (3.75)	1426 (4.23)	113 (3.05)	116 (3.09)	389 (6.84)	2239 (5.39)	307 (3.18)	1144 (3.55)	299 (3.51)
Missing	496 (0.08)	95 (0.09)	24 (0.12)	37 (0.11)	2 (0.05)	4 (0.11)	4 (0.07)	29 (0.07)	11 (0.11)	29 (0.09)	11 (0.13)
Marital status											
Unmarried	589 988 (90.77)	93 100 (87.19)	16 699 (85.02)	28 928 (85.87)	3227 (87.21)	3332 (88.78)	4947 (87.03)	36 240 (87.18)	8431 (87.29)	27 137 (84.17)	7162 (84.03)
Married	59 389 (9.14)	13 621 (12.76)	2936 (14.95)	4754 (14.11)	472 (12.76)	419 (11.16)	729 (12.83)	5293 (12.73)	1222 (12.65)	5093 (15.80)	1356 (15.91)
Missing	579 (0.09)	56 (0.05)	6 (0.03)	5 (0.01)	1 (0.03)	2 (0.05)	8 (0.14)	37 (0.09)	6 (0.06)	12 (0.04)	5 (0.06)
Country of birth											
Finland	607 366 (93.45)	99 015 (92.73)	18 891 (96.18)	32 190 (95.56)	3345 (90.41)	3587 (95.58)	5087 (89.50)	36 783 (88.48)	8981 (92.98)	30 799 (95.52)	8095 (94.98)
Not Finland	42 590 (6.55)	7762 (7.27)	750 (3.82)	1497 (4.44)	355 (9.59)	166 (4.42)	597 (10.50)	4787 (11.52)	678 (7.02)	1443 (4.48)	428 (5.02)
Socioeconomic status											
Upper white collar	110 603 (17.02)	14 065 (13.17)	2502 (12.74)	4733 (14.05)	572 (15.46)	627 (16.71)	557 (9.80)	4283 (10.30)	1428 (14.78)	3329 (10.33)	1131 (13.27)
Lower white collar	210 070 (32.32)	32 633 (30.56)	6047 (30.79)	10 170 (30.19)	1139 (30.78)	1256 (33.47)	1696 (29.84)	12 302 (29.59)	2964 (30.69)	9717 (30.14)	2440 (28.63)
Blue collar	82 574 (12.70)	16 677 (15.62)	3281 (16.70)	5378 (15.96)	475 (12.84)	473 (12.60)	1045 (18.38)	7136 (17.17)	1319 (13.66)	5442 (16.88)	1292 (15.16)
Missing or undefined	246 709 (37.96)	43 402 (40.65)	7811 (39.77)	13 406 (39.80)	1514 (40.92)	1397 (37.22)	2386 (41.98)	17 849 (42.94)	3948 (40.87)	13 754 (42.66)	3660 (42.94)
Smoking											
No	536 070 (82.48)	80 075 (75.00)	13 716 (69.83)	24 380 (72.37)	2934 (79.30)	2984 (79.51)	4293 (75.53)	30 874 (74.27)	7561 (78.28)	21 863 (67.81)	6146 (72.11)
Continued	98 189 (15.11)	24 001 (22.48)	5416 (27.57)	8474 (25.16)	680 (18.38)	695 (18.52)	1213 (21.34)	9594 (23.08)	1869 (19.35)	8109 (25.15)	2182 (25.60)
Missing	15 697 (2.42)	2701 (2.53)	509 (2.59)	833 (2.47)	86 (2.32)	74 (1.97)	178 (3.13)	1102 (2.65)	229 (2.37)	755 (2.34)	195 (2.29)
Maternal inpatient & outpatient psychiatric disorder, during pregnancy											
No	641 457 (98.69)	90 169 (84.45)	19 049 (96.99)	32 555 (96.64)	3576 (96.65)	3620 (96.46)	5561 (97.84)	40 565 (97.58)	9305 (96.34)	31 074 (96.38)	8124 (95.32)
Yes	8499 (1.31)	16 608 (15.55)	592 (3.01)	1132 (3.36)	124 (3.35)	133 (3.54)	123 (2.16)	1005 (2.42)	354 (3.66)	1168 (3.62)	399 (4.68)
Maternal inpatient & outpatient psychiatric disorder, before pregnancy											
No	585 596 (90.10)	103 870 (97.28)	16 456 (83.78)	28 023 (83.19)	3106 (83.95)	3104 (82.71)	4838 (85.12)	35 059 (84.34)	8003 (82.86)	25 877 (80.26)	6690 (78.49)
Yes	64 360 (9.90)	2907 (2.72)	3185 (16.22)	5664 (16.81)	594 (16.05)	649 (17.29)	846 (14.88)	6511 (15.66)	1656 (17.14)	6365 (19.74)	1833 (21.51)
Maternal systemic inflammatory disorder											
No	644 323 (99.13)	105 827 (99.11)	19 630 (99.94)	33 669 (99.95)	n >5	3748 (99.87)	n >5	41 550 (99.95)	9650 (99.91)	32 221 (99.93)	n >5
Yes	5633 (0.87)	950 (0.89)	11 (0.06)	18 (0.05)	n >5	5 (0.13)	n >5	20 (0.05)	9 (0.09)	21 (0.07)	n >5
Maternal diabetes											
No	544 748 (83.81)	86 640 (81.14)	15 768 (80.28)	6086 (18.06)	2982 (80.59)	3135 (83.53)	4486 (78.92)	33 014 (79.42)	7764 (80.38)	26 103 (80.96)	6951 (81.56)
Yes	105 208 (16.19)	20 137 (18.86)	3873 (19.72)	27 601 (81.93)	718 (19.41)	618 (16.47)	1198 (21.08)	8556 (20.58)	1895 (19.62)	6139 (19.04)	1572 (18.44)
Adverse birth outcomes^b^											
Gestational age at delivery, wk											
22-31	5056 (0.78)	1694 (1.59)	129 (0.66)	287 (0.85)	171 (4.62)	39 (1.04)	197 (3.47)	1128 (2.71)	139 (1.44)	436 (1.35)	108 (1.27)
32-36	31 126 (4.79)	6494 (6.08)	888 (4.52)	1692 (5.02)	350 (9.46)	283 (7.54)	501 (8.81)	3023 (7.27)	562 (5.82)	1920 (5.95)	469 (5.50)
37-41	581 568 (89.48)	93 137 (87.23)	17 595 (89.58)	29 964 (88.95)	3023 (81.70)	3263 (86.94)	4720 (83.04)	35 344 (85.02)	8434 (87.32)	28 227 (87.55)	7502 (88.02)
≥42	30 627 (4.71)	5123 (4.80)	962 (4.90)	1625 (4.82)	149 (4.03)	156 (4.16)	241 (4.24)	1952 (4.70)	485 (5.02)	1561 (4.84)	410 (4.81)
Missing	1579 (0.24)	329 (0.31)	67 (0.34)	119 (0.35)	7 (0.19)	12 (0.32)	25 (0.44)	123 (0.30)	39 (0.40)	98 (0.30)	34 (0.40)
Birth weight for gestational age											
Small	21 181 (3.26)	5138 (4.81)	758 (3.86)	1331 (3.95)	388 (10.49)	140 (3.73)	600 (10.56)	2517 (6.05)	461 (4.77)	1557 (4.83)	378 (4.44)
Appropriate	609 050 (93.71)	98 213 (91.98)	18 266 (93.00)	31 220 (92.68)	3184 (86.05)	3477 (92.65)	4877 (85.80)	37 611 (90.48)	8911 (92.26)	29 713 (92.16)	7877 (92.42)
Large	17 654 (2.72)	3077 (2.88)	541 (2.75)	994 (2.95)	115 (3.11)	111 (2.96)	172 (3.03)	1264 (3.04)	242 (2.51)	851 (2.64)	229 (2.69)
Missing	2071 (0.32)	439 (0.41)	76 (0.39)	142 (0.42)	13 (0.35)	25 (0.67)	35 (0.62)	178 (0.43)	45 (0.47)	121 (0.38)	39 (0.46)
Apgar score 1 min											
0-6	36 596 (5.63)	7712 (7.22)	1087 (5.53)	2000 (5.94)	413 (11.16)	279 (7.43)	773 (13.60)	3653 (8.79)	772 (7.99)	2281 (7.07)	56 (0.66)
7-10	611 756 (94.12)	98 721 (92.46)	18 516 (94.27)	31 591(93.78)	3271 (88.41)	3460 (92.19)	4875 (85.77)	37 744 (90.80)	8853 (91.66)	29 853 (92.60)	7926 (93.00)
Missing	1604 (0.25)	344 (0.32)	38 (0.19)	96 (0.28)	16 (0.43)	14 (0.37)	36 (0.63)	173 (0.42)	34 (0.35)	108 (0.33)	29 (0.34)
Apgar score 5 min											
0-6	12 374 (1.90)	2880 (2.70)	344 (1.75)	644 (1.91)	184 (4.97)	94 (2.50)	373 (6.56)	1566 (3.77)	295 (3.05)	818 (2.54)	200 (2.35)
7-10	538 106 (82.79)	86 294 (80.82)	15 689 (79.88)	27 108 (80.47)	2912 (78.70)	3012 (80.26)	4550 (80.05)	33 056 (79.52)	7818 (80.94)	26 295 (81.56)	6795 (79.73)
Missing	99 476 (15.31)	17 603 (16.49)	3608 (18.37)	5935 (17.62)	604 (16.32)	647 (17.24)	761 (13.39)	6948 (16.71)	1546 (16.01)	5129 (15.91)	1528 (17.93)
Head circumference, cm											
<32	16 816 (2.59)	4342 (4.07)	444 (2.26)	951 (2.82)	351 (9.49)	144 (3.84)	516 (9.08)	2407 (5.79)	366 (3.79)	1179 (3.66)	288 (3.38)
≥32	604 870 (93.06)	95 765 (89.69)	17 221 (87.68)	29 989 (89.02)	3129 (84.57)	3397 (90.51)	4764 (83.81)	36 733 (88.36)	8713 (90.21)	29 284 (90.83)	7722 (90.60)
Missing	28 270 (4.35)	6670 (6.25)	1976 (10.06)	2747 (8.15)	220 (5.95)	212 (5.65)	401 (7.05)	2430 (5.85)	580 (6.00)	1779 (5.52)	513 (6.02)

Abbreviations: ADHD, attention-deficit/hyperactivity disorder; AN, anorexia nervosa; ASD, autism spectrum disorders; BMI, body mass index (calculated as weight in kilograms divided by height in meters squared); BN, bulimia nervosa; EDNOS, eating disorders not otherwise specified.

^a^ Total case numbers of the cohort and the diagnostic categories for offspring psychiatric disorders.

^b^ Frequencies are represented as case numbers and percentages and are represented in 3 categories: exposures, covariates, and adverse birth outcomes.

^c^ BMI categories: underweight, less than 18.5; normal weight, 18.5 to 24.9; overweight, 25.0 to 29.9; obesity, 30.0 to 34.9; and severe obesity, 35.0 or higher.

### Statistical Analysis

We used Cox proportional hazards modeling to obtain hazard ratios (HRs) with 95% Wald CIs of the associations for maternal eating disorders and prepregnancy BMI with risks of offspring psychiatric diagnoses by evaluating the exposure to their respective reference being mothers without an eating disorder diagnosis or mothers with normal weight. The obtained crude estimates (nonadjusted model) were subsequently adjusted for covariates in the 2 aforementioned models. The proportional hazards assumptions were tested. To address the potential bias introduced by mothers having more than 1 child, we performed a sensitivity analysis including only the firstborn offspring of each mother. To address the association with adverse birth outcomes, we performed a sensitivity analysis stratifying for offspring with at least 1 of the following: gestational age at delivery less than 37 weeks, small birth weight for gestational age (below 2 SDs of the mean adjusted for sex and gestational age), minimum Apgar score of 6 or less at 1 and 5 minutes, or head circumference 32 cm or less. We also performed a sensitivity analysis excluding offspring older than 10 years with an eating disorder diagnosis (ever). We used SAS, version 9.4 (SAS Institute Inc), for the statistical analysis. To control for type 1 errors, the corrected statistical significance threshold was set at a 2-sided *P* ≤ .005 (Bonferroni correction for multiple testing based on 10 offspring outcome diagnoses).

## Results

### Study Population

The mean (SD) age of 392 098 included mothers was 30.15 (5.38) years, 42 590 (10.86%) mothers were born outside of Finland, 6273 mothers (1.60%) had a history of eating disorders, 23 114 mothers (5.89%) had prepregnancy underweight, and a total of 208 335 (53.13%) mothers had overweight or obesity (134 584 [34.32%] had overweight, 49 909 [12.73%] had obesity, and 23 842 [6.08%] had severe obesity). Among 649 956 included offspring, 317 597 (48.86%) were female and 332 359 (51.14%) were male. From birth until 7 to 17 years of age, 106 777 (16.43%) offspring were diagnosed with a neurodevelopmental or psychiatric disorder. Of those offspring, 19 641 (3.02%) were diagnosed with mood disorder, 33 687 (5.18%) with anxiety disorder, 3700 (0.57%) with other feeding disturbances in infancy and childhood, 3753 (0.58%) with sleep disorders, 5684 (0.87%) with intellectual disabilities, 41 570 (6.40%) with specific developmental disorders, 9659 (1.49%) with ASD, 32 242 (4.96%) with ADHD or conduct disorders, and 8523 (1.31%) with social functioning and tic disorders. Frequencies of exposures and covariates are given in the Table. The rate of missing data for each exposure and covariate in the total cohort was lower than 1%, except for maternal prepregnancy BMI (range, 5.18%-11.65%), socioeconomic status (range, 37.96%-42.94%), 5-minute Apgar score (range, 13.39%-18.37%), and head circumference (range, 4.35%-10.06%).

### Maternal Eating Disorders and Offspring Psychiatric Diagnoses

**Figure 1.**
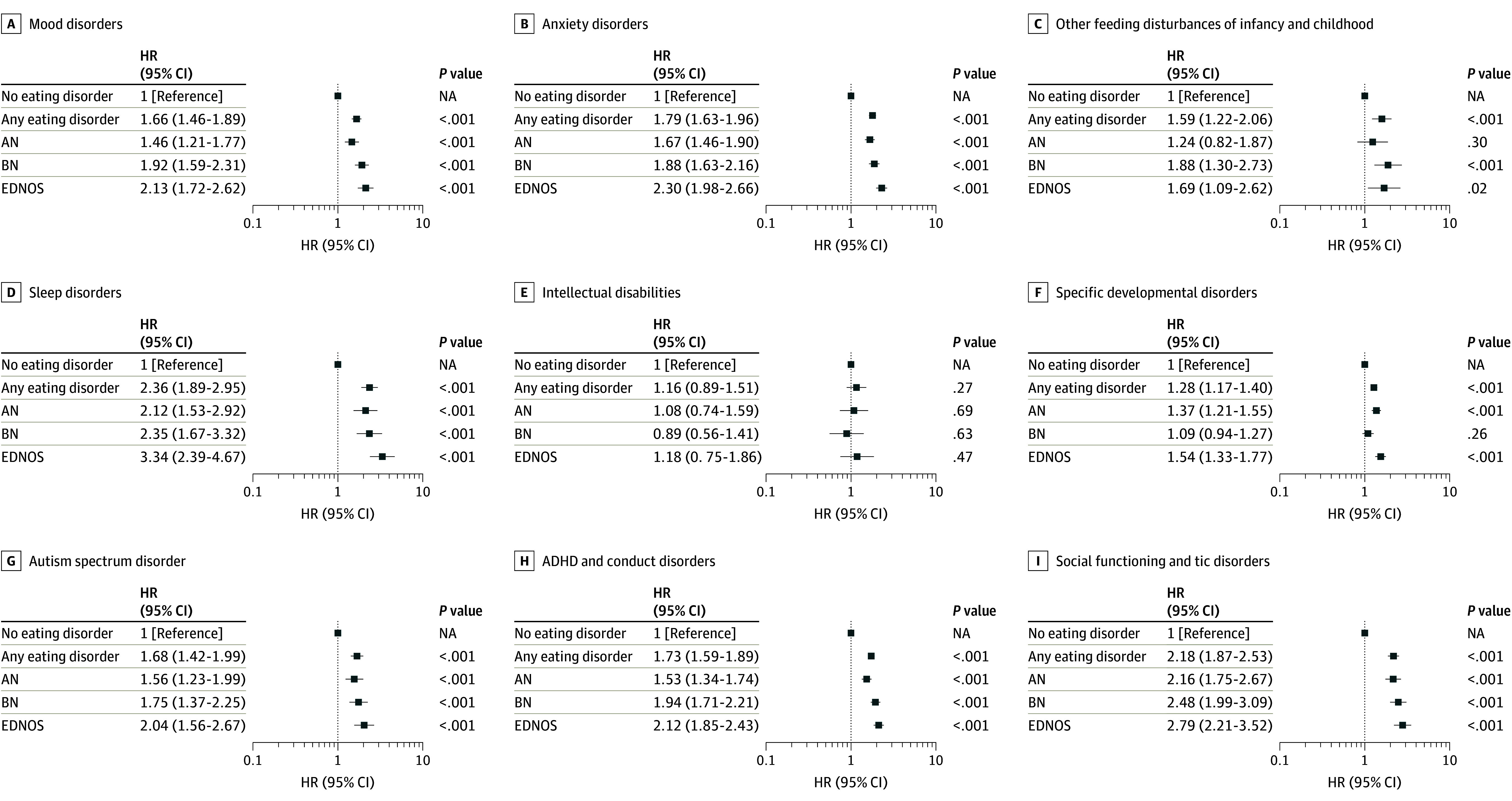
Associations Between Maternal Eating Disorders and Offspring Psychiatric Diagnoses The hazard ratios (HRs) are from adjusted model 2. Numbers in each subgroup are provided in the main text and Table. Analyses were adjusted for offspring birth year and sex, number of fetuses, maternal age group at delivery, parity, unmarried mother at birth, mother’s country of birth, maternal smoking, maternal socioeconomic status, maternal prepregnancy body mass index, maternal systemic and bowel inflammatory disorders, and maternal diabetes. ADHD indicates attention-deficit/hyperactivity disorder; AN, anorexia nervosa; BN, bulimia nervosa; EDNOS, eating disorder not otherwise specified; NA, not applicable.

We detected associations of maternal eating disorders, combined and separated into AN, BN, and EDNOS, with all analyzed offspring psychiatric diagnoses except intellectual disabilities (eTable 3 in Supplement 1; Figure 1). All associations remained after adjusting for covariates in model 2, except maternal EDNOS with offspring other feeding disturbances in childhood and infancy. There was no association of maternal AN with offspring ASD at the corrected level in the crude model, but there was an association in the adjusted models. The largest effect sizes in the most adjusted model were observed for sleep disorders (HR, 2.36 [95% CI, 1.89-2.95] for maternal eating disorders; HR, 2.12 [95% CI, 1.53-2.92] for AN; HR, 2.35 [95% CI, 1.67-3.32] for BN; and HR, 3.34 [95% CI, 2.39-4.67] for EDNOS) and social functioning and tic disorders (HR, 2.18 [95% CI, 1.87-2.53] for eating disorders; HR, 2.16 [95% CI, 1.75-2.67] for AN; HR, 2.48 [95% CI, 1.99-3.09] for BN; and HR, 2.79 [95% CI, 2.21-3.52] for EDNOS), with more than a doubled risk for offspring of mothers with eating disorders. The results were verified using only the firstborn offspring of each mother. When stratified for adverse birth outcomes, we found even higher effect sizes of most exposures on offspring diagnoses (eTable 2 in Supplement 1). A markedly higher HR was observed for offspring feeding disturbances in infancy and childhood in offspring with both maternal eating disorders and adverse birth outcome (HR, 4.53 [95% CI, 2.97-6.89] for eating disorders; HR, 4.30 [95% CI, 2.38-7.78] for AN; HR, 4.46 [95% CI, 2.32-8.58] for BN; and HR, 4.25 [95% CI, 2.03-8.94] for EDNOS), while maternal AN and EDNOS without adverse birth outcomes were not associated with this group of other pediatric feeding disorders (HR, 1.40 [95% CI, 1.00-1.96] for eating disorders; HR, 0.93 [95% CI, 0.53-1.64] for AN; and HR, 1.58 [95% CI, 0.92-2.73] for EDNOS). Similarly, the effect sizes of AN in offspring with adverse birth outcomes on ADHD and conduct disorders were higher than that of either of these 2 exposures (HR, 2.27 [95% CI, 1.74-2.96] for AN). The sensitivity analysis excluding offspring with comorbid eating disorders showed that most associations remained, with no considerable change in effect size (eTable 3 in Supplement 1). The association between maternal BN and offspring sleep disorders, however, did not remain (HR, 2.35 [95% CI, 1.67-3.32] for BN in the full cohort; HR, 1.84 [95% CI, 1.13-3.01] for BN excluding offspring with eating disorders).

### Maternal Prepregnancy BMI and Offspring Psychiatric Diagnoses

**Figure 2.**
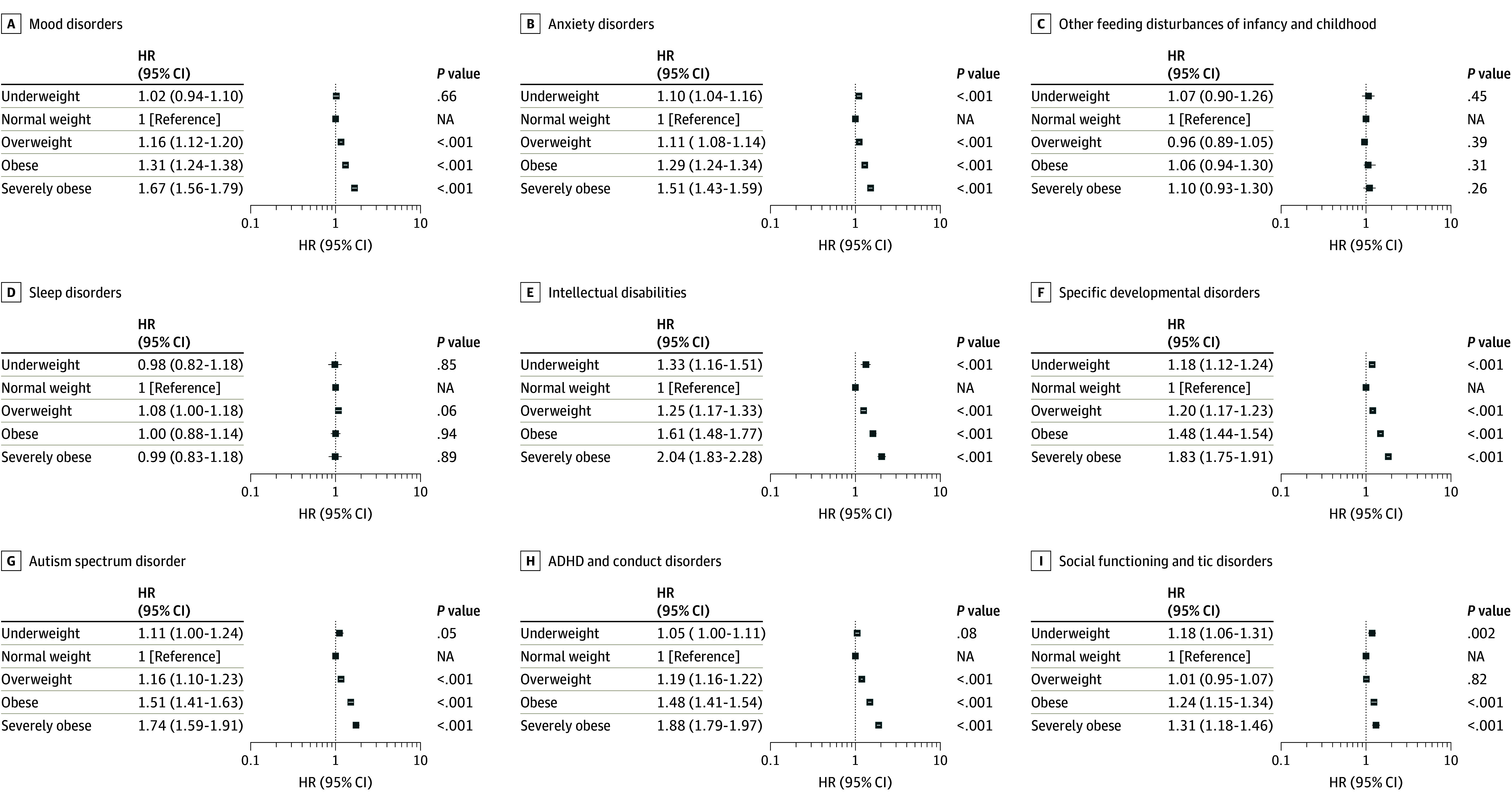
Associations Between Maternal Body Mass Index (BMI) Category and Offspring Psychiatric Diagnoses Categories for BMI (calculated as weight in kilograms divided by height in meters squared): underweight, less than 18.5; normal weight, 18.5 to 24.9; overweight, 25.0 to 29.0; obesity, 30.0 to 34.0; and severe obesity, 35.0 or higher. Numbers in each subgroup are provided in the main text and Table. Analyses were adjusted for offspring birth year and sex, number of fetuses, maternal age group at delivery, parity, unmarried mother at birth, mother’s country of birth, maternal smoking, maternal socioeconomic status, maternal inpatient or outpatient psychiatric disorder, maternal systemic inflammatory disorder, and maternal diabetes. ADHD indicates attention-deficit/hyperactivity disorder; HR, hazard ratio; NA, not applicable.

We detected associations between maternal prepregnancy underweight for all analyzed offspring psychiatric diagnoses except for other feeding disturbances of infancy and childhood and for sleep disorders (eTable 4 in Supplement 1; Figure 2). After adjusting for covariates, the associations remained, but had small effect sizes for anxiety (HR, 1.10 [95% CI, 1.04-1.16]), intellectual disabilities (HR, 1.33 [95% CI, 1.16-1.51]), specific developmental disorders (HR, 1.18 [95% CI, 1.12-1.24]), and social functioning and tic disorders (HR, 1.18 [95% CI, 1.06-1.31]). For maternal prepregnancy overweight and obesity, we detected associations in adjusted model 2 for most offspring psychiatric diagnoses except for other feeding disturbances in infancy and childhood and for sleep disorders, with higher effect sizes with increasing BMI (ie, overweight < obesity < severe obesity). The largest effect sizes were observed for intellectual disabilities (HR, 1.25 [95% CI, 1.17-1.33] for overweight; HR, 1.61 [95% CI, 1.48-1.77] for obesity; and HR, 2.04 [95% CI, 1.83-2.28] for severe obesity). The results were verified using only the firstborn offspring of each mother. Offspring exposed to both higher BMI and adverse birth outcome had higher risk for specific developmental disorders and ADHD and conduct disorder than offspring exposed to only 1 of these exposures (eTable 5 in Supplement 1). With adverse birth outcomes, we noted associations between maternal underweight, normal weight, or overweight or obesity and offspring other feeding disturbances in infancy and childhood, as well as sleep disorders, not observed without such complications (eTable 5 in Supplement 1).

## Discussion

In this cohort study, we found associations of maternal eating disorders, AN, BN, and EDNOS, as well as prepregnancy BMI outside normal weight, with several neurodevelopmental and psychiatric diagnoses in offspring, even after adjusting for covariates. We found significantly increased risks of all studied offspring neurodevelopmental and psychiatric diagnoses, except intellectual disabilities, associated with the various maternal eating disorders. However, we could not detect any association of maternal AN with offspring other feeding disturbances in childhood and infancy, nor maternal BN with offspring specific developmental disorders. The largest effect sizes were observed for offspring sleep disorders and social functioning and tic disorders, with a more than doubled risk by each maternal eating disorder, with maternal EDNOS showing the highest effect size. This study is, to our knowledge, the first time these associations have been reported. Maternal eating disorders, both active and previous, have been associated with offspring cord blood DNA methylation, in particular in loci relevant to metabolism,^43^ and this association may be part of the mechanism behind the associations observed here. Other potential mechanisms could be related to nutritional deficiencies as a result of restricted eating and specific dietary regimens common with eating disorders.^7^

Since maternal history of AN worsens the risk of adverse birth outcomes in offspring,^10,44,45^ we stratified the analyses for prematurity, low Apgar score, and abnormal size at birth. We detected a markedly higher risk for other feeding disturbances of infancy and childhood and of ADHD and conduct disorders among offspring of mothers with a history of eating disorders in the group who reported such events at birth compared with no such events. Some adverse birth outcomes have been reported to be associated with feeding and eating disturbances in children aged 0 to 3 years.^46^ Brosig et al^47^ stated that birth-related complications may be associated with avoidant restrictive food intake disorder, introduced in the *Diagnostic and Statistical Manual of Mental Disorders* (Fifth Edition). That study highlighted the importance of studying perinatal risk factors of this specific and related eating disorders, which is supported by our findings. One speculation is that adverse birth outcomes, such as prematurity, delay the onset of independent oral feeding, which could influence coordination of oral sensorimotor systems and the tuning of neuronal circuitries regulating food intake. We also find it interesting that the effect sizes of maternal AN in offspring with adverse birth outcomes on offspring ADHD and conduct disorders were higher than that of either of these 2 exposures alone, requiring further investigation.

Multimorbidity of psychiatric disorders is common,^48^ not the least of which is comorbidity of eating disorders with other psychiatric disorders.^49^ In addition, additive genetic factors account for approximately 40% to 60% of the liability associated with eating disorders,^50^ and a high degree of genetic correlation between psychiatric disorders is documented.^51^ Aiming to control for the genetics of eating disorders, we performed an analysis stratifying the offspring cohort by the presence or absence of a comorbid eating disorder diagnosis, with maternal eating disorders as the exposure. No marked changes were detected in risks between the 2 groups except for reduced risk of offspring sleep disorders with maternal BN. Thus, the majority of associations observed here may be speculated to not be the result of shared genetics.

With maternal underweight, we found associations with anxiety disorder, intellectual disabilities, specific developmental disorders, and social functioning and tic disorders, after adjusting for covariates. With maternal overweight or obesity, associations were detected with all included diagnoses except for sleep disorders and for other feeding disturbances of childhood and infancy. The effect sizes were smaller in general for maternal BMI compared with those for eating disorders. As outlined in the Introduction, both maternal underweight or starvation^26,27,28,29,30,31^ and overweight or obesity have previously been associated with offspring psychiatric disorders,^20,21,22,23,24^ and our study adds to this line of report. As already mentioned, maternal underweight can be accompanied with deficiencies in micronutrients and macronutrients of importance for the developing brain, but overweight is also associated with micronutrient deficiencies in particular.^52^ In addition, obesity is associated with low grade inflammation, hyperglycemia, insulin resistance, and high circulating leptin, all of which can influence neuronal development.^34,53,54,55,56,57^ Overweight and obesity are associated with birth complications and deviations.^58^ In general, when stratifying for adverse birth outcomes, the effect size increases, in particular for underweight as well as for overweight or obesity, for specific developmental disorders were higher than that of either of these exposures alone. A similar result was also observed for overweight or obesity with adverse birth outcomes and ADHD and conduct disorder. In addition, we observed associations for all maternal BMI categories with other feeding disturbances in childhood and infancy in the group that experienced complications, strengthening the argument that adverse birth outcomes may be associated with these pediatric conditions.

We find it interesting that the results for maternal underweight and AN were not the same. For example, while maternal AN was associated with offspring sleep disorders, maternal underweight was not. On the contrary, maternal underweight was associated with offspring intellectual disabilities, while maternal AN was not. We controlled for maternal BMI in the adjusted models exploring the association between maternal eating disorder and offspring mental disorders. This finding suggests that the association between maternal AN and the mentioned offspring diagnoses is not mediated by underweight in general but rather the specific underweight in AN or some other accompanying trait, for example, a dietary regimen, extreme physical activity, or endocrine abnormalities.^59^ Further studies exploring the mechanisms behind this association will be valuable.

### Strengths and Limitations

A major strength of this study is the large cohort size resulting from the use of population-based Finnish registers. The Finnish health care system is based on a public system striving to provide equal access for all to health care. The results presented here should thus represent the Finnish population at large, even if skewness in health care consumption cannot be excluded.

A limitation is the relatively short follow-up time (7-17 years of age) in relation to some of the late-onset psychiatric diagnoses. This limitation restricted us from including schizophrenia spectrum disorders and personality disorders (eTable 6 in Supplement 1 for estimated proportion of identified cases). Even though we included many variables of good quality,^60,61^ paternal data and genetic information were not available. A potential bias specific for the association between maternal history of eating disorders with other feeding disturbances in childhood and infancy is that mothers with eating disorders may be more perceptive to their child’s eating behavior, resulting in higher access to care and diagnosis for these children. However, such an association was not observed with maternal AN, which speaks against this speculation.

## Conclusions

The findings of this cohort study suggest that offspring born to mothers with eating disorders before or during pregnancy, or who had prepregnancy underweight, overweight, or obesity, may be at higher risk of neurodevelopmental and psychiatric disorders, indicating a need to consider these exposures clinically to help prevent offspring mental illness. The offspring diagnoses associated with each of the exposures differed somewhat, with effect sizes typically larger for maternal eating disorders than for maternal BMI outside normal weight. These associations were also present among offspring who did not experience adverse birth outcomes, but when the exposures were combined with adverse birth outcomes, a higher risk of a psychiatric disorder diagnosis was typically observed. This was particularly pronounced for other feeding disorders in infancy and childhood, specific developmental disorders, and ADHD and conduct disorder, highlighting the need to explore the association between these diagnoses and adverse birth outcomes. Furthering the knowledge about these associations and underlying biological mechanisms can provide information of value for the development of relevant management and treatment.

## Notes

Supplement 1. **eTable 1.** Hazard ratios (HR) for offspring psychiatric and mild neurodevelopmental diagnoses in relation to maternal eating disorders (n = 649956) followed until 2021 **eTable 2.** Hazard ratios (HR) for offspring psychiatric and mild neurodevelopmental diagnoses in relation to maternal eating disorders stratified for adverse birth outcomes, n_wo adverse birth outcomes_ = 564173, n_w adverse birth outcomes_ = 85783, followed until 2021 **eTable 3.** Hazard ratios (HR) for offspring psychiatric and mild neurodevelopmental diagnosis in relation to maternal eating disorders excluding offspring with comorbid eating disorders, n_full cohort_ = n = 649956, n_excl. offspring eating disorders_ = 470983, followed until 2021 **eTable 4.** Hazard ratios (HR) for offspring psychiatric and mild neurodevelopmental diagnosis in relation to maternal prepregnancy body mass index (n = 649956) followed until 2021 **eTable 5.** Hazard ratios (HR) for offspring psychiatric and mild neurodevelopmental diagnosis in relation to maternal prepregnancy BMI stratified for adverse birth outcomes, n_wo adverse birth outcomes_ = 564173, n_w adverse birth outcomes_ = 85783, followed until 2021 **eTable 6.** List of neurodevelopmental and psychiatric diagnoses along with ICD-10 codes, and the corresponding proportions that were identified (until 2021) out of the estimated number of cases that would have received a diagnosis before 17 years of age (requiring the youngest cases to be followed up until 2031

Supplement 2.
Data Sharing Statement
