# Supplementary material for: Maternal Eating Disorders, Body Mass Index, and Offspring Psychiatric Diagnoses
Source: JAMA Netw Open. 2024 Oct 22;7(10):e2440517. doi: 10.1001/jamanetworkopen.2024.40517 (PMC11581519; doi:10.1001/jamanetworkopen.2024.40517)
Supplement: Supplement 1. — eTable 1. Hazard ratios (HR) for offspring psychiatric and mild neurodevelopmental diagnoses in relation to maternal eating disorders (n = 649956) followed until 2021 eTable 2. Hazard ratios (HR) for offspring psychiatric and mild neurodevelopmental diagnoses in relation to maternal eating disorders stratified for adverse birth outcomes, nwo adverse birth outcomes = 564173, nw adverse birth outcomes = 85783, followed until 2021 eTable 3. Hazard ratios (HR) for offspring psychiatric and mild neurodevelopmental diagnosis in relation to maternal eating disorders excluding offspring with comorbid eating disorders, nfull cohort = n = 649956, nexcl. offspring eating disorders = 470983, followed until 2021 eTable 4. Hazard ratios (HR) for offspring psychiatric and mild neurodevelopmental diagnosis in relation to maternal prepregnancy body mass index (n = 649956) followed until 2021 eTable 5. Hazard ratios (HR) for offspring psychiatric and mild neurodevelopmental diagnosis in relation to maternal prepregnancy BMI stratified for adverse birth outcomes, nwo adverse birth outcomes = 564173, nw adverse birth outcomes = 85783, followed until 2021 eTable 6. List of neurodevelopmental and psychiatric diagnoses along with ICD-10 codes, and the corresponding proportions that were identified (until 2021) out of the estimated number of cases that would have received a diagnosis before 17 years of age (requiring the youngest cases to be followed up until 2031 [file jamanetwopen-e2440517-s001.pdf]

Supplementary Online Content

Nilsson IAK, Ozsvar J, Gissler M, Lavebratt C. Maternal eating disorders, body mass index and offspring psychiatric diagnoses. *JAMA Netw Open*. 2024;7(10):e2440517. doi:10.1001/jamanetworkopen.2024.40517

eTable 1. Hazard ratios (HR) for offspring psychiatric and mild neurodevelopmental diagnoses in relation to maternal eating disorders (n= 649956) followed until 2021
eTable 2. Hazard ratios (HR) for offspring psychiatric and mild neurodevelopmental diagnoses in relation to maternal eating disorders stratified for adverse birth outcomes, $n_{\text{wo adverse birth outcomes}}$ = 564173, $n_{\text{w adverse birth outcomes}}$ = 85783, followed until 2021
eTable 3. Hazard ratios (HR) for offspring psychiatric and mild neurodevelopmental diagnosis in relation to maternal eating disorders excluding offspring with comorbid eating disorders, $n_{\text{full cohort}}$= n= 649956, $n_{\text{excl. offspring eating disorders}}$= 470983, followed until 2021
eTable 4. Hazard ratios (HR) for offspring psychiatric and mild neurodevelopmental diagnosis in relation to maternal prepregnancy body mass index (n=649956) followed until 2021
eTable 5. Hazard ratios (HR) for offspring psychiatric and mild neurodevelopmental diagnosis in relation to maternal prepregnancy BMI stratified for adverse birth outcomes, $n_{\text{wo adverse birth outcomes}}$ = 564173, $n_{\text{w adverse birth outcomes}}$ = 85783, followed until 2021
eTable 6. List of neurodevelopmental and psychiatric diagnoses along with ICD-10 codes, and the corresponding proportions that were identified (until 2021) out of the estimated number of cases that would have received a diagnosis before 17 years of age (requiring the youngest cases to be followed up until 2031

This supplementary material has been provided by the authors to give readers additional information about their work.

© 2024 Nilsson IAK et al. *JAMA Network Open*.

eTable 1. Hazard ratios (HR) for offspring psychiatric and mild neurodevelopmental diagnoses in relation to maternal eating disorders (n= 649956) followed until 2021

Abbreviations: ADHD, attention deficit hyperactivity disorder; AN, anorexia nervosa; ASD, autism spectrum disorders; BN, bulimia nervosa; CI, confidence interval; EDNOS, eating disorders not otherwise specified; HR, hazard ratios; NA, not applicable.

Offspring diagnoses were F30-39, F92 (mood disorders), F40-43, F93 (anxiety disorders), F98.2 (other feeding disturbances of infancy & childhood), F51 (sleeping disorders), F70-79 (intellectual disabilities), F80-83 (specific developmental disorders), F84 (ASD), F90-91 (ADHD and conduct disorders) and F94-95 (social functioning and tic disorders). Maternal diagnoses were No eating disorders (all N excluding mother's with F50, 3071A, 3075B or 3075E), Any eating disorders (F50, 3071A, 3075B or 3075E), AN (F50.0, F50.1, 3071A), BN (F50.2, F50.3, 3071B), EDNOS (F50.8, F50.9, 3071E).

Cohort 2004-2014, n=649956. All were followed up until end of 2021.

Reference: births to mothers without a history of eating disorders. Bold numbers represent p<0.05, * p<0.005.

[a]Not adjusted

[b]Model 1a: the analyses were adjusted for offspring birth year, sex, number of fetuses, maternal age group at delivery, parity, unmarried mother at birth, mother's country of birth, maternal smoking, maternal SES, maternal prepregnancy BMI.

[c]Model 2a: the analyses were adjusted for model 1 variables plus maternal systemic and bowel inflammatory disorders (M30-M36, K50, K51, K52.3) and maternal diabetes (E10, E14, O24.1, O24.4).

**Offspring Psychiatric diagnoses:**	**Any F (N=106777)**	**Mood disorders (N=19641)**	**Anxiety disorders (N=33687)**	**Other feeding disturbances of infancy & childhood (N=3700)**	**Sleep disorders (N=3753)**	**Intellectual disabilities (N=5684)**	**Specific developmental disorders (N=41570)**	**ASD (N=9659)**	**ADHD and conduct disorders (N=32242)**	**Social functioning and tic disorders (N=8523)**
	HR (95% CI)	HR (95% CI)	HR (95% CI)	HR (95% CI)	HR (95% CI)	HR (95% CI)	HR (95% CI)	HR (95% CI)	HR (95% CI)	HR (95% CI)
**Categories of maternal eating disorders[a]: Crude model[a]**										
No	1.00 (NA)	1.00 (NA)	1.00 (NA)	1.00 (NA)	1.00 (NA)	1.00 (NA)	1.00 (NA)	1.00 (NA)	1.00 (NA)	1.00 (NA)

© 2024 Nilsson IAK et al. *JAMA Network Open*.

Any	**1.42 (1.35-1.50)***	**1.30 (1.14-1.48)***	**1.50 (1.37-1.64)***	**1.62 (1.25-2.10)***	**2.27 (1.81-2.83)***	1.05 (0.81-1.36)	**1.22 (1.11-1.34)***	**1.57 (1.33-1.86)***	**1.76 (1.61-1.92)***	**2.22 (1.91-2.60)***
AN	**1.30 (1.21-1.41)***	1.11 (0.91-1.34)	**1.36 (1.19-1.55)***	1.25 (0.83-1.88)	**2.01 (1.46-2.78)***	0.93 (0.63-1.37)	**1.24 (1.10-1.41)***	**1.41 (1.11-1.80)**	**1.48 (1.30-1.68)***	**2.14 (1.74-2.64)***
BN	**1.48 (1.36-1.60)***	**1.52 (1.26-1.84)***	**1.58 (1.38-1.82)***	**1.94 (1.34-2.81)***	**2.30 (1.64-3.24)***	0.82 (0.52-1.30)	1.05 (0.91-1.23)	**1.70 (1.32-2.18)***	**2.00 (1.76-2.27)***	**2.53 (2.03-3.15)***
EDNOS	**1.71 (1.57-1.86)***	**1.62 (1.31-2.00)***	**1.90 (1.64-2.20)***	**1.78 (1.15-2.76)**	**3.18 (2.28-4.44)***	1.13 (0.72-1.77)	**1.58 (1.37-1.83)***	**1.92 (1.47-2.52)***	**2.33 (2.04-2.67)***	**2.99 (2.37-3.77)***
**Model 1a**[b]										
No	1.00 (NA)	1.00 (NA)	1.00 (NA)	1.00 (NA)	1.00 (NA)	1.00 (NA)	1.00 (NA)	1.00 (NA)	1.00 (NA)	1.00 (NA)
Any	**1.51 (1.43-1.59)***	**1.66 (1.46-1.89)***	**1.79 (1.63-1.96)***	**1.59 (1.22-2.07)***	**2.36 (1.89-2.95)***	1.16 (0.88-1.50)	**1.28 (1.17-1.40)***	**1.68 (1.42-1.99)***	**1.73 (1.59-1.89)***	**2.18 (1.87-2.53)***
AN	**1.44 (1.34-1.55)***	**1.46 (1.20-1.77)***	**1.67 (1.46-1.90)***	1.24 (0.82-1.87)	**2.12 (1.53-2.93)***	1.08 (0.73-1.59)	**1.37 (1.21-1.55)***	**1.56 (1.23-1.99)***	**1.53 (1.34-1.74)***	**2.16 (1.75-2.67)***
BN	**1.56 (1.44-1.69)***	**1.92 (1.59-2.31)***	**1.87 (1.63-2.16)***	**1.87 (1.29-2.72)***	**2.35 (1.67-3.32)***	0.89 (0.56-1.42)	1.09 (0.93-1.27)	**1.75 (1.37-2.25)***	**1.94 (1.71-2.21)***	**2.48 (1.99-3.09)***
EDNOS	**1.76 (1.61-1.91)***	**2.13 (1.73-2.63)***	**2.30 (1.98-2.66)***	**1.69 (1.09-2.63)**	**3.36 (2.40-4.69)***	1.18 (0.75-1.86)	**1.54 (1.33-1.77)***	**2.04 (1.56-2.68)***	**2.13 (1.86-2.44*)**	**2.79 (2.21-3.52)***
**Model 2a**[c]										
No	1.00 (NA)	1.00 (NA)	1.00 (NA)	1.00 (NA)	1.00 (NA)	1.00 (NA)	1.00 (NA)	1.00 (NA)	1.00 (NA)	1.00 (NA)
Any	**1.51 (1.44-1.60)***	**1.66 (1.46-1.89)***	**1.79 (1.63-1.96)***	**1.59 (1.22-2.06)***	**2.36 (1.89-2.95)***	1.16 (0.89-1.51)	**1.28 (1.17-1.40)***	**1.68 (1.42-1.99)***	**1.73 (1.59-1.89)***	**2.18 (1.87-2.53)***

© 2024 Nilsson IAK et al. *JAMA Network Open*.

AN	**1.44 (1.34-1.55)***	**1.46 (1.21-1.77)***	**1.67 (1.46-1.90)***	1.24 (0.82-1.87)	**2.12 (1.53-2.92)***	1.08 (0.74-1.59)	**1.37 (1.21-1.55)***	**1.56 (1.23-1.99)***	**1.53 (1.34-1.74)***	**2.16 (1.75-2.67)***
BN	**1.56 (1.44-1.69)***	**1.92 (1.59-2.31)***	**1.88 (1.63-2.16)***	**1.88 (1.30-2.73)***	**2.35 (1.67-3.32)***	0.89 (0.56-1.41)	1.09 (0.94-1.27)	**1.75 (1.37-2.25)***	**1.94 (1.71-2.21)***	**2.48 (1.99-3.09)***
EDNOS	**1.75 (1.61-1.91)***	**2.13 (1.72-2.62)***	**2.30 (1.98-2.66)***	**1.69 (1.09-2.62)**	**3.34 (2.39-4.67)***	1.18 (0.75-1.86)	**1.54 (1.33-1.77)***	**2.04 (1.56-2.67)***	**2.12 (1.85-2.43)***	**2.79 (2.21-3.52)***

© 2024 Nilsson IAK et al. *JAMA Network Open*.

eTable 2. Hazard ratios (HR) for offspring psychiatric and mild neurodevelopmental diagnoses in relation to maternal eating disorders stratified for adverse birth outcomes, $n_{wo\ adverse\ birth\ outcomes}$ = 564173, $n_{w\ adverse\ birth\ outcomes}$ = 85783, followed until 2021

Abbreviations: ADHD, attention deficit hyperactivity disorder; adv. birth, adverse birth outcomes; AN, anorexia nervosa; ASD, autism spectrum disorders; BN, bulimia nervosa; CI, confidence interval; EDNOS, eating disorders not otherwise specified; HR, hazard ratios; NA, not applicable wo, without.
Offspring diagnoses were F30-39, F92 (mood disorders), F40-43, F93 (anxiety disorders), F98.2 (other feeding disturbances of infancy & childhood), F51 (sleep disorders), F70-79 (intellectual disabilities), F80-83 (specific developmental disorders), F84 (ASD), F90-91 (ADHD and conduct disorders) and F94-95 (social functioning and tic disorders). Maternal diagnoses were No eating disorders (all N excluding mother's with F50, 3071A, 3075B or 3075E), Any eating disorders (F50, 3071A, 3075B or 3075E), AN (F50.0, F50.1, 3071A), BN (F50.2, F50.3, 3071B), EDNOS (F50.8, F50.9, 3071E).
Adverse birth outcomes were defined a gestational age at delivery ≤ week 36, small birth weight for gestational age, Apgar score ≤ 6, and/or head circumference ≤ 32 cm. Cohort 2004-2014, $n_{wo\ adverse\ birth\ outcomes}$ = 564173, $n_{w\ adverse\ birth\ outcomes}$ = 85783, All were followed up until end of 2021.
Reference: births to mothers without a history of eating disorders. Bold numbers represent p<0.05,* p<0.005.
Model 2a: the analyses were adjusted for offspring birth year, sex, number of fetuses, maternal age group at delivery, parity, unmarried mother at birth, mother's country of birth, maternal smoking, maternal SES, maternal prepregnancy BMI, maternal systemic and bowel inflammatory disorders (M30-M36, K50, K51, K52.3) and maternal diabetes (E10, E14, O24.1, O24.4).

**Offspring psychiatric diagnoses:**	**Any F (N=106777)**	**Mood disorders (N=19641)**	**Anxiety disorders (N=33687)**	**Other feeding disturbances of infancy & childhood (N=3700)**	**Sleep disorders (N=3753)**	**Intellectual disabilities (N=5684)**	**Specific developmental disorders (N=41570)**	**ASD (N=9659)**	**ADHD and conduct disorders (N=32242)**	**Social functioning and tic disorders (N=8523)**
	HR (95% CI)	HR (95% CI)	HR (95% CI)	HR (95% CI)	HR (95% CI)	HR (95% CI)	HR (95% CI)	HR (95% CI)	HR (95% CI)	HR (95% CI)
**Categories of maternal eating disorders[a]:**										
**Model 2a[a]**										
**No**										

© 2024 Nilsson IAK et al. *JAMA Network Open*.

wo adv.birth.	1.00 (NA)	1.00 (NA)	1.00 (NA)	1.00 (NA)	1.00 (NA)	1.00 (NA)	1.00 (NA)	1.00 (NA)	1.00 (NA)	1.00 (NA)
adv.birth.	**1.35 (1.33-1.37)***	**1.05 (1.01-1.10)**	**1.13 (1.09-1.17)***	**2.43 (2.25-2.62)***	**1.30 (1.19-1.42)***	**2.67 (2.52-2.83)***	**1.62 (1.58-1.66)***	**1.37 (1.30-1.45)***	**1.28 (1.25-1.33)***	**1.23 (1.15-1.30)***
**Any**										
wo adv.birth	**1.53 (1.44-1.62)***	**1.64 (1.42-1.89)***	**1.78 (1.61-1.97)***	**1.40 (1.00-1.96)**	**2.44 (1.91-3.12)***	1.22 (0.89-1.66)	**1.31 (1.18-1.45)***	**1.72 (1.43-2.07)***	**1.72 (1.56-1.89)***	**2.23 (1.89-2.63)***
adv.birth	**1.89 (1.68-2.13)***	**1.84 (1.35-2.51)***	**2.05 (1.65-2.55)***	**4.53 (2.97-6.89)***	**2.56 (1.51-4.30)***	**2.50 (1.53-4.09)***	**1.83 (1.51-2.22)***	**2.02 (1.37-2.97)***	**2.22 (1.84-2.69)***	**2.32 (1.61-3.35)***
**AN**										
wo adv.birth	**1.45 (1.33-1.57)***	**1.38 (1.11-1.72)***	**1.61 (1.39-1.87)***	0.93 (0.53-1.64)	**2.16 (1.50-3.09)***	0.91 (0.55-1.51)	**1.42 (1.23-1.63)***	**1.48 (1.12-1.96)**	**1.46 (1.25-1.69)***	**2.12 (1.68-2.69)***
adv.birth	**1.84 (1.55-2.18)***	**1.93 (1.27-2.94)***	**2.14 (1.59-2.88)***	**4.30 (2.38-7.78)***	**2.40 (1.14-5.04)***	**3.49 (1.93-6.30)***	**1.86 (1.42-2.44)***	**2.47 (1.51-4.04)***	**2.27 (1.74-2.96)***	**2.75 (1.73-4.37)***
**BN**										
wo adv.birth	**1.58 (1.45-1.73)***	**1.97 (1.61-2.41)***	**1.90 (1.63-2.21)***	**1.84 (1.17-2.89)**	**2.42 (1.65-3.53)***	1.03 (0.61-1.74)	1.08 (0.90-1.28)	**1.90 (1.45-2.49)***	**1.98 (1.71-2.28)***	**2.62 (2.07-3.32)***
adv.birth	**1.90 (1.58-2.29)***	**1.75 (1.07-2.86)**	**1.97 (1.39-2.78)***	**4.46 (2.32-8.58)***	**2.62 (1.17-5.83)***	1.44 (0.54-3.84)	**1.71 (1.27-2.32)***	1.59 (0.83-3.06)	**2.22 (1.67-2.96)***	**2.24 (1.27-3.94)***
**EDNOS**										
wo adv.birth	**1.79 (1.63-1.97)***	**2.13 (1.69-2.68)***	**2.28 (1.94-2.69)***	1.58 (0.92-2.73)	**3.51 (2.44-5.07)***	1.38 (0.83-2.30)	**1.62 (1.38-1.90)***	**2.06 (1.52-2.79)***	**2.14 (1.84-2.49)***	**2.93 (2.27-3.77)***
adv.birth.	**2.08 (1.71-2.54)***	**2.19 (1.32-3.63)***	**2.56 (1.81-3.62)***	**4.25 (2.03-8.94)***	**3.36 (1.51-7.47)***	1.82 (0.68-4.86)	**1.95 (1.42-2.68)***	**2.59 (1.43-4.67)***	**2.56 (1.89-3.46)***	**2.61 (1.45-4.72)***

© 2024 Nilsson IAK et al. *JAMA Network Open*.

eTable 3. Hazard ratios (HR) for offspring psychiatric and mild neurodevelopmental diagnosis in relation to maternal eating disorders excluding offspring with comorbid eating disorders, $n_{full\ cohort}$= n= 649956, $n_{excl.\ offspring\ eating\ disorders}$= 470983, followed until 2021

Abbreviations: ADHD, attention deficit hyperactivity disorder; AN, anorexia nervosa; Any eating disorder, any eating disorder; ASD, autism spectrum disorders; BN, bulimia nervosa; CI, confidence interval; EDNOS, eating disorders not otherwise specified; HR, hazard ratios; NA, not applicable.
Offspring diagnoses were F30-39, F92 (mood disorders), F40-43, F93 (anxiety disorders), F98.2 (other feeding disturbances of infancy & childhood), F51 (sleep disorders), F70-79 (intellectual disabilities), F80-83 (specific developmental disorders), F84 (ASD), F90-91 (ADHD and conduct disorders) and F94-95 (social functioning and tic disorders). Maternal diagnoses were No eating disorders (all N excluding mother's with F50, 3071A, 3075B or 3075E), Any eating disorder (F50, 3071A, 3075B or 3075E), AN (F50.0, F50.1, 3071A), BN (F50.2, F50.3, 3071B), EDNOS (F50.8, F50.9, 3071E).
Cohort 2004-2014, $n_{full\ cohort}$= 649956, $n_{excl.\ offspring\ eating\ disorders}$ = 470983, All were followed up until end of 2021.
Reference: births to mothers without a history of eating disorders. Bold numbers represent p<0.05,* p<0.005.
Model 2a: the analyses were adjusted for offspring birth year, sex, number of fetuses, maternal age group at delivery, parity, unmarried mother at birth, mother's country of birth, maternal smoking, maternal SES, maternal prepregnancy BMI, maternal systemic and bowel inflammatory disorders (M30-M36, K50, K51, K52.3) and maternal diabetes (E10, E14, O24.1, O24.4)

© 2024 Nilsson IAK et al. *JAMA Network Open*.

**Offspring psychiatric diagnoses:**	**Any F (N=106777/83162)**	**Mood disorders (N=19641/17685)**	**Anxiety disorders (N=33687/29559)**	**Other feeding disturbances of infancy & childhood (N=3700/2603)**	**Sleep disorders (N=3753/2804)**	**Intellectual disabilities (N=5684/4474)**	**Specific developmental disorders (N=41570/31132)**	**ASD (N=9659/7692)**	**ADHD and conduct disorders (N=32242/25969)**	**Social functioning and tic disorders (N=8523/6871)**
	HR (95% CI)	HR (95% CI)	HR (95% CI)	HR (95% CI)	HR (95% CI)	HR (95% CI)	HR (95% CI)	HR (95% CI)	HR (95% CI)	HR (95% CI)
**Categories of maternal eating disorders [a]: Model 2a[a]**										
**No**										
Full cohort	1.00 (NA)	1.00 (NA)	1.00 (NA)	1.00 (NA)	1.00 (NA)	1.00 (NA)	1.00 (NA)	1.00 (NA)	1.00 (NA)	1.00 (NA)
**Any**										
Full cohort	**1.51 (1.44-1.60)***	**1.66 (1.46-1.89)***	**1.79 (1.63-1.96)***	**1.59 (1.22-2.06)***	**2.36 (1.89-2.95)***	1.16 (0.89-1.51)	**1.28 (1.17-1.40)***	**1.68 (1.42-1.99)***	**1.73 (1.59-1.89)***	**2.18 (1.87-2.53)***
Excl. offspring eating disorders	**1.54 (1.44-1.64)***	**1.67 (1.45-1.92)***	**1.84 (1.66-2.04)***	**1.72 (1.24-2.38)***	**2.17 (1.62-2.92)***	1.21 (0.89-1.66)	**1.27 (1.14-1.43)***	**1.81 (1.49-2.19)***	**1.73 (1.56-1.92)***	**2.11 (1.76-2.53)***
**AN**										
Full cohort	**1.44 (1.34-1.55)***	**1.46 (1.21-1.77)***	**1.67 (1.46-1.90)***	1.24 (0.82-1.87)	**2.12 (1.53-2.92)***	1.08 (0.74-1.59)	**1.37 (1.21-1.55)***	**1.56 (1.23-1.99)***	**1.53 (1.34-1.74)***	**2.16 (1.75-2.67)***
Excl. offspring eating disorders	**1.46 (1.33-1.60)***	**1.49 (1.21-1.83)***	**1.70 (1.47-1.97)***	1.27 (0.75-2.15)	**1.86 (1.20-2.90)**	1.22 (1.79-1.90)	**1.38 (1.18-1.61)***	**1.62 (1.22-2.15)***	**1.51 (1.29-1.76)***	**2.00 (1.54-2.59)***
**BN**										
Full cohort	**1.56 (1.44-1.69)***	**1.92 (1.59-2.31)***	**1.88 (1.63-2.16)***	**1.88 (1.30-2.73)***	**2.35 (1.67-3.32)***	0.89 (0.56-1.41)	1.09 (0.94-1.27)	**1.75 (1.37-2.25)***	**1.94 (1.71-2.21)***	**2.48 (1.99-3.09)***

© 2024 Nilsson IAK et al. *JAMA Network Open*.

Excl. offspring eating disorders	**1.56 (1.41-1.72)***	**1.87 (1.52-2.30)***	**1.90 (1.63-2.22)***	**2.12 (1.35-3.33)***	**1.84 (1.13-3.01)**	0.58 (0.29-1.16)	0.99 (0.81-1.21)	**1.77 (1.31-2.37)***	**1.93 (1.65-2.24)***	**2.58 (2.00-3.33)***
**EDNOS**										
Full cohort	**1.75 (1.61-1.91)***	**2.13 (1.72-2.62)***	**2.30 (1.98-2.66)***	**1.69 (1.09-2.62)**	**3.34 (2.39-4.67)***	1.18 (0.75-1.86)	**1.54 (1.33-1.77)***	**2.04 (1.56-2.67)***	**2.12 (1.85-2.43)***	**2.79 (2.21-3.52)***
Excl. offspring eating disorders	**1.71 (1.53-1.91)***	**2.09 (1.66-2.63)***	**2.27 (1.92-2.69)***	1.40 (0.72-2.69)	**3.61 (2.35-5.56)***	1.40 (0.82-2.33)	**1.52 (1.26-1.83)***	**2.36 (1.73-3.22)***	**1.99 (1.68-2.37)***	**2.36 (1.73-3.22)***

© 2024 Nilsson IAK et al. *JAMA Network Open*.

eTable 4. Hazard ratios (HR) for offspring psychiatric and mild neurodevelopmental diagnosis in relation to maternal prepregnancy body mass index (n=649956) followed until 2021

Abbreviations: ADHD, attention deficit hyperactivity disorder; ASD, autism spectrum disorders; CI; confidence interval; HR, hazard ratios; NA, not applicable.

Offspring diagnoses were F30-39, F92 (mood disorders), F40-43, F93 (anxiety disorders), F98.2 (other feeding disturbances of infancy and childhood), F51 (sleep disorders), F70-79 (intellectual disabilities), F80-83 (specific developmental disorders), F84 (ASD), F90-91 (ADHD and conduct disorders) and F94-95 (social functioning and tic disorders).

Cohort 2004-2014, n=649956. All were followed up until end of 2021.

Reference: births to normal weight mothers (BMI 18.5-24). Bold numbers represent p<0.05,* p<0.005.

[a]Not adjusted

[b]Model 1b: the analyses were adjusted for offspring birth year, sex, number of fetuses, maternal age group at delivery, parity, unmarried mother at birth, mother's country of birth, maternal smoking, maternal SES**,** maternal in- or outpatient psychiatric disorder in mothers (ICD-8: 290-317; ICD-9: 290-319; ICD-10: F00-F99).

[c]Model 2b: the analyses were adjusted for model 1 variables plus maternal systemic inflammatory disorder (M30-M36, K50, K51, K52.3) and maternal diabetes (E10, E14, O24.1, O24.4).

© 2024 Nilsson IAK et al. *JAMA Network Open*.

Offspring psychiatric diagnoses:	**Any F (N=106777)**	**Mood disorders (N=19641)**	**Anxiety disorders (N=33687)**	**Other feeding disturbances of infancy & childhood (N=3700)**	**Sleep disorders (N=3753)**	**Intellectual disabilities (N=5684)**	**Specific developmental disorders (N=41570)**	**ASD (N=9659)**	**ADHD and conduct disorders (N=32242)**	**Social functioning and tic disorders (N=8523)**
	HR (95% CI)	HR (95% CI)	HR (95% CI)	HR (95% CI)	HR (95% CI)	HR (95% CI)	HR (95% CI)	HR (95% CI)	HR (95% CI)	HR (95% CI)
**Categories of body mass index [kg/m²][a]:**										
**Crude model[a]**										
underweight	**1.26 (1.23-1.30)***	**1.25 (1.16-1.35)***	**1.29 (1.22-1.36)***	1.15 (0.97-1.35)	1.01 (0.85-1.21)	**1.52 (1.33-1.72)***	**1.39 (1.33-1.46)***	**1.22 (1.10-1.36)***	**1.31 (1.24-1.39)***	**1.40 (1.26-1.55)***
normal weight	1.00 (NA)	1.00 (NA)	1.00 (NA)	1.00 (NA)	1.00 (NA)	1.00 (NA)	1.00 (NA)	1.00 (NA)	1.00 (NA)	1.00 (NA)
overweight	**1.15 (1.14-1.17)***	**1.18 (1.14-1.23)***	**1.12 (1.09-1.15)***	0.99 (0.91-1.08)	**1.09 (1.01-1.19)**	**1.31 (1.23-1.40)***	**1.25 (1.22-1.29)***	**1.18 (1.12-1.24)***	**1.22 (1.18-1.25)***	1.03 (0.98-1.09)
obese	**1.38 (1.35-1.41)***	**1.37 (1.30-1.44)***	**1.32 (1.27-1.37)***	**1.14 (1. 01-1.28)**	1.03 (0.91-1.16)	**1.77 (1.62-1.92)***	**1.63 (1.57-1.68)***	**1.55 (1.45-1.66)***	**1.57 (1.52-1.63)***	**1.32 (1.23-1.43)***
severely obese	**1.65 (1.51-1.59)***	**1.76 (1.65-1.88)***	**1.56 (1.48-1.64)***	**1.22 (1.04-1.43)**	1.03 (0.86-1.23)	**2.31 (2.07-2.56)***	**2.08 (1.45-1.57)***	**1.83 (1.68-2.00)***	**2.08 (1.99-2.18)***	**1.46 (1.32-1.62)***
**Model 1b[b]**										
underweight	**1.10 (1.06-1.13)***	1.01 (0.94-1.09)	**1.10 (1.04-1.16)***	1.06 (0.90-1.25)	0.98 (0.82-1.18)	**1.32 (1.16-1.50)***	**1.17 (1.12-1.23)***	1.11 (1.00-1.23)	1.05 (0.99-1.11)	**1.18 (1.06-1.31)***
normal weight	1.00 (NA)	1.00 (NA)	1.00 (NA)	1.00 (NA)	1.00 (NA)	1.00 (NA)	1.00 (NA)	1.00 (NA)	1.00 (NA)	1.00 (NA)
overweight	**1.15 (1.13-1.17)***	**1.19 (1.15-1.24)***	**1.12 (1.09-1.16)***	1.00 (0.92-1.08)	**1.09 (1.00-1.18)**	**1.27 (1.19-1.36)***	**1.23 (1.20-1.26)***	**1.19 (1.13-1.25)***	**1.21 (1.17-1.24)***	1.03 (0.97-1.09)
obese	**1.36 (1.33-1.39)***	**1.38 (1.31-1.45)***	**1.32 (1.27-1.37)***	**1.14 (1.01-1.28)**	1.01 (0.89-1.15)	**1.68 (1.54-1.84)***	**1.56 (1.52-1.61)***	**1.57 (1.47-1.68)***	**1.52 (1.46-1.58)***	**1.29 (1.20-1.39)***

© 2024 Nilsson IAK et al. *JAMA Network Open*.

severely obese	**1.61 (1.57-1.65)***	**1.81 (1.69-1.93)***	**1.56 (1.48-1.64)***	**1.21 (1.03-1.43)**	1.00 (0.84-1.19)	**2.18 (1.96-2.42)***	**1.97 (1.89-2.05)***	**1.83 (1.68-2.00)***	**1.95 (1.87-2.05)***	**1.39 (1.25-1.54)***
**Model 2b[c]**										
underweight	**1.10 (1.07-1.14)***	1.02 (0.94-1.10)	**1.10 (1.04-1.16)***	1.07 (0.90-1.26)	0.98 (0.82-1.18)	**1.33 (1.16-1.51)***	**1.18 (1.12-1.24)***	1.11 (1.00-1.24)	1.05 (1.00-1.11)	**1.18 (1.06-1.31)***
normal weight	1.00 (NA)	1.00 (NA)	1.00 (NA)	1.00 (NA)	1.00 (NA)	1.00 (NA)	1.00 (NA)	1.00 (NA)	1.00 (NA)	1.00 (NA)
overweight	**1.13 (1.11-1.14)***	**1.16 (1.12-1.20)***	**1.11 (1.08-1.14)***	0.96 (0.89-1.05)	1.08 (1.00-1.18)	**1.25 (1.17-1.33)***	**1.20 (1.17-1.23)***	**1.16 (1.10-1.23)***	**1.19 (1.16-1.22)***	1.01 (0.95-1.07)
obese	**1.31 (1.28-1.34)***	**1.31 (1.24-1.38)***	**1.29 (1.24-1.34)***	1.06 (0.94-1.30)	1.00 (0.88-1.14)	**1.61 (1.48-1.77)***	**1.48 (1.44-1.54)***	**1.51 (1.41-1.63)***	**1.48 (1.41-1.54)***	**1.24 (1.15-1.34)***
severely obese	**1.53 (1.49-1.57)***	**1.67 (1.56-1.79)***	**1.51 (1.43-1.59)***	1.10 (0.93-1.30)	0.99 (0.83-1.18)	**2.04 (1.83-2.28)***	**1.83 (1.75-1.91)***	**1.74 (1.59-1.91)***	**1.88 (1.79-1.97)***	**1.31 (1.18-1.46)***

© 2024 Nilsson IAK et al. *JAMA Network Open*.

eTable 5. Hazard ratios (HR) for offspring psychiatric and mild neurodevelopmental diagnosis in relation to maternal prepregnancy BMI stratified for adverse birth outcomes, $n_{wo\ adverse\ birth\ outcomes}$ = 564173, $n_{w\ adverse\ birth\ outcomes}$ = 85783, followed until 2021

Abbreviations: ADHD, attention deficit hyperactivity disorder; adv. birth, adverse birth outcomes; ASD, autism spectrum disorders; BN, bulimia nervosa; CI, confidence interval; HR, hazard ratios; NA, not applicable.; wo, without.
Offspring diagnoses were F30-39, F92 (mood disorders), F40-43, F93 (anxiety disorders), F98.2 (other feeding disturbances of infancy and childhood), F51 (sleep disorders), F70-79 (intellectual disabilities), F80-83 (specific developmental disorders), F84 (ASD), F90-91 (ADHD and conduct disorders) and F94-95 (social functioning and tic disorders).
Adverse birth outcomes were defined a gestational age at delivery ≤ week 36, small birth weight for gestational age, Apgar score ≤ 6, head circumference ≤ 32 cm.
Cohort 2004-2014, $n_{wo\ adverse\ birth\ outcomes}$ = 564173, $n_{w\ adverse\ birth\ outcomes}$ = 85783.All were followed up until end of 2021
Reference: births to normal weight mothers (BMI 18.5-24). Bold numbers represent p<0.05,* p<0.005.
[a]Model 2b: the analyses were adjusted for offspring birth year, sex, number of fetuses, maternal age group at delivery, parity, unmarried mother at birth, mother's country of birth, maternal smoking, maternal SES**,** maternal in- or outpatient psychiatric disorder in mothers (ICD-8: 290-317; ICD-9: 290-319; ICD-10: F00-F99), maternal systemic inflammatory disorder (M30-M36, K50, K51, K52.3) and maternal diabetes (E10, E14, O24.1, O24.4).

**Offspring Psychiatric diagnoses:**	**Any F (N=106777)**	**Mood disorders (N=19641)**	**Anxiety disorders (N=33687)**	**Other feeding disturbances of infancy & childhood (N=3700)**	**Sleep disorders (N=3753)**	**Intellectual disabilities (N=5684)**	**Specific developmental disorders (N=41570)**	**ASD (N=9659)**	**ADHD and conduct disorders (N=32241)**	**Social functioning and tic disorders (N=8523)**
	HR (95% CI)	HR (95% CI)	HR (95% CI)	HR (95% CI)	HR (95% CI)	HR (95% CI)	HR (95% CI)	HR (95% CI)	HR (95% CI)	HR (95% CI)
**Categories of body mass index [kg/m^2][a]: Model 2b[a] underweight**										
wo adv.birth.	**1.06 (1.02-1.10)***	0.93 (0.86-1.01)	1.06 (0.99-1.12)	1.02 (0.83-1.25)	1.00 (0.82-1.21)	**1.33 (1.14-1.55)***	**1.12 (1.06-1.19)***	1.08 (0.95-1.21)	1.05 (0.98-1.11)	1.12(1.00-1.26)

© 2024 Nilsson IAK et al. *JAMA Network Open*.

adv.birth.	**1.44 (1.34-1.54)***	1.18 (1.00-1.40)	**1.23 (1.08-1.39)***	**2.45 (1.83-3.26)***	1.00 (0.66-1.52)	**2.93 (2.33-3.69)***	**1.78 (1.61-1.96)***	**1.53 (1.23-1.91)***	**1.21 (1.07-1.38)***	**1.50 (1.21-1.87)***
**normal weight**										
wo adv.birth.	1.00 (NA)	1.00 (NA)	1.00 (NA)	1.00 (NA)	1.00 (NA)	1.00 (NA)	1.00 (NA)	1.00 (NA)	1.00 (NA)	1.00 (NA)
adv.birth.	**1.32 (1.29-1.35)***	0.98 (0.92-1.04)	**1.09 (1.04-1.14)***	**2.32 (2.10-2.55)***	**1.22 (1.09-1.37)***	**2.77 (2.56-3.01)***	**1.59 (1.54-1.65)***	**1.37 (1.28-1.48)***	**1.29 (1.24-1.35)***	**1.19 (1.10-1.29)***
**overweight**										
wo adv.birth.	**1.10 (1.08-1.12)***	**1.10 (1.06-1.14)***	**1.08 (1.05-1.11)***	0.91 (0.82-1.00)	1.03 (0.94-1.13)	**1.25 (1.15-1.35)***	**1.17 (1.13-1.20)***	**1.15 (1.09-1.22)***	**1.17 (1.14-1.21)***	0.99 (0.93-1.05)
adv.birth.	**1.47 (1.42-1.52)***	**1.15 (1.06-1.14)***	**1.21 (1.13-1.29)***	**2.55 (2.22-2.93)***	**1.48 (1.26-1.75)***	**3.15 (2.81-3.53)***	**1.78 (1.70-1.87)***	**1.54 (1.38-1.72)***	**1.49 (1.40-1.58)***	**1.16 (1.02-1.32)**
**obese**										
wo adv.birth.	**1.28 (1.25-1.32)***	**1.26 (1.19-1.33)***	**1.26 (1.21-1.32)***	1.06 (0.92-1.22)	0.97 (0.84-1.12)	**1.66 (1.50-1.84)***	**1.44 (1.39-1.49)***	**1.52 (1.38-1.72)***	**1.47 (1.41-1.53)***	**1.24 (1.14-1.35)***
adv.birth..	**1.66 (1.58-1.74)***	**1.20 (1.04-1.37)**	**1.31 (1.19-1.45)***	**2.39 (1.92-2.96)***	1.30 (1.00-1.70)	**3.71 (3.17-4.34)***	**2.22 (2.08-2.37)***	**1.84 (1.58-2.15)***	**1.74 (1.60-1.90)***	**1.29 (1.07-1.55)**
**severely**										
wo adv.birth..	**1.49 (1.44-1.54)***	**1.57 (1.46-1.69)***	**1.46 (1.38-1.54)***	1.17 (0.97-1.42)	0.99 (0.81-1.20)	**2.05 (1.80-2.33)***	**1.79 (1.71-1.88)***	**1.76 (1.59-1.95)***	**1.86 (1.77-1.96)***	**1.33 (1.19-1.49)***
adv.birth.	**1.92 (1.80-2.04)***	**1.70 (1.45-1.98)***	**1.65 (1.46-1.86)***	**1.94 (1.41-2.66)***	1.09 (0.73-1.62)	**4.66 (3.87-5.63)***	**2.55 (2.34-2.78)***	**1.98 (1.62-2.42)**	**2.24 (2.02-2.48)***	**1.28 (1.00-1.65)**

© 2024 Nilsson IAK et al. *JAMA Network Open*.

eTable 6. List of neurodevelopmental and psychiatric diagnoses along with *ICD-10* codes, and the corresponding proportions that were identified (until 2021) out of the estimated number of cases that would have received a diagnosis before 17 years of age (requiring the youngest cases to be followed up until 2031)

Abbreviations: ASD, autism spectrum disorders; and ADHD, attention-deficit/hyperactivity disorders. The Finnish Care Registers for Health Care (HILMO) contains data on all hospital in-patient treatments (since 1969) as well as out-patient treatments by physicians in specialized care (since 1998) and includes all psychiatric diagnoses.
Estimated proportion identified was calculated as number of diagnosed cases divided by the denominator being: (proportion of diagnosed cases among those born in 2004 (this birth cohort was followed up for 17 years)*total cohort size 649956).

**Neurodevelopmental and psychiatric disorders of interest**	***ICD-10* codes**	***Estimated proportion identified (%)***
Any neurodevelopmental and psychiatric disorders	F00-F99	
Psychotic disorders	F20-F29	30.4
Mood disorders	F30-F39 and F92	36.6
Anxiety disorders	F40-F43 and F93	46.2
Eating disorders	F50	35.0
Sleep disorders	F51	66.0
Personality disorders	F60-F69	35.0
Intellectual disabilities	F70-F79	76.9
Specific developmental disorders	F80-F83	92.5
ASD	F84	77.2
ADHD and conduct disorders	F90	83.4
Social functioning and tic disorders	F94-95	86.9
Other feeding disturbances of infancy & childhood	F98.2	113.4

© 2024 Nilsson IAK et al. *JAMA Network Open*.
