# Supplementary material for: Maternal Eating Disorders, Body Mass Index, and Offspring Psychiatric Diagnoses
Source: JAMA Netw Open. 2024 Oct 22;7(10):e2440517. doi: 10.1001/jamanetworkopen.2024.40517 (PMC11581519; doi:10.1001/jamanetworkopen.2024.40517)
Supplement: Supplement 2. — Data Sharing Statement [file jamanetwopen-e2440517-s002.pdf]

# Data Sharing Statement

Nilsson. Maternal Eating Disorders, Body Mass Index, and Offspring Psychiatric Diagnoses. *JAMA Netw Open*. Published October 22, 2024. doi:10.1001/jamanetworkopen.2024.40517

**Data**
**Data available:** No

**Additional Information**
**Explanation for why data not available:** The data supporting this study's findings are available from the Finnish Institute for Health and Welfare and Social Insurance Institution. Restrictions apply to the availability of these data, which were used under license for this specific study. Similar data are available with the permission of the Finnish Social and Health Data Permit Authority Findata.
